# Insula deep rTMS and varenicline for smoking cessation: A randomized control trial study protocol

**DOI:** 10.3389/fphar.2022.969500

**Published:** 2022-09-08

**Authors:** Christine Ibrahim, Saima Malik, Mera S. Barr, Daniel M. Blumberger, Zafiris J. Daskalakis, Bernard Le Foll

**Affiliations:** ^1^ Translational Addiction Research Laboratory, Centre for Addiction and Mental Health, Toronto, ON, Canada; ^2^ Department of Pharmacology and Toxicology, Faculty of Medicine, University of Toronto, Toronto, ON, Canada; ^3^ Canadian Institutes of Health Research, Ottawa, ON, Canada; ^4^ Temerty Centre for Therapeutic Brain Intervention, Centre of Addiction and Mental Health, Toronto, ON, Canada; ^5^ Department of Psychiatry, Faculty of Medicine, University of Toronto, Toronto, ON, Canada; ^6^ Department of Psychiatry, School of Medicine, University of California, San Diego Health, San Diego, CA, United States; ^7^ Centre for Addiction and Mental Health, Campbell Family Mental Health Research Institute, Toronto, ON, Canada; ^8^ Centre for Addiction and Mental Health, Addictions Division, Toronto, ON, Canada; ^9^ Institute of Medical Sciences, University of Toronto, Toronto, ON, Canada; ^10^ Department of Family and Community Medicine, University of Toronto, Toronto, ON, Canada

**Keywords:** deep repetitive transcranial magnetic stimulation (dTMS), varenicline, smoking cesation, insula, protocol, RCT, randomized controlled trial

## Abstract

**Background:** Current approved therapies for smoking cessation have modest long-term effects for abstinence. The insular cortex has been identified by preclinical and clinical studies as a critical target for addiction treatment. Insula functions can be modulated non-invasively using brain stimulation. It is unknown if deep repetitive transcranial magnetic stimulation (rTMS) of the insula can improve smoking cessation of smokers trying to quit using varenicline.

**Methods:** This will be a randomized, double-blind, sham-controlled clinical trial with 50 nicotine dependent smokers looking to quit. They will be randomly assigned to receive either active (10 Hz) or sham insula deep rTMS. Deep rTMS will be administered for 4 weeks (5 days/week). All participants will receive open label varenicline for 12 weeks. The primary outcome measure will be the 7-day point prevalence abstinence at the end of 12 weeks. The secondary outcomes will be Fagerström Test of Nicotine Dependence, Minnesota Nicotine Withdrawal Scale, Tiffany Questionnaire of Smoking Urges, expired carbon monoxide measurements, cigarettes smoked per day, point prevalence abstinence at end of 4 weeks, prolonged and continuous abstinence at 6 months. The measures will be collected throughout the 3-month treatment period as well as at the 6-month follow up.

**Discussion:** This trial will test for the first time the impact of deep insula rTMS on smoking cessation in smokers treated with varenicline. This trial will use an H-coil specific to the insula, while previous studies have targeted both the insula and prefrontal cortex. This trial will inform on the utility to combine insula deep rTMS with varenicline to improve smoking abstinence rates.

**Clinical Trial Registration:** Trial registered at https://clinicaltrials.gov/ct2/show/NCT04083144 (Identifier: NCT04083144).

## 1 Introduction

Tobacco it is still one of the largest public health threats and leading causes of death worldwide (Le Foll et al., 2022). There are approximately 1.3 billion smokers in the world and tobacco kills more than eight million people each year (World Health Organization, 2021). Currently, there are three approved pharmacotherapies for smoking cessation: Varenicline, bupropion, and nicotine replacement therapy. These therapies have shown efficacy but only short-term, with trials showing that nearly three quarters of the quitters relapse within a year (Hajek et al., 2009; Hajek et al., 2013). Smokers with psychiatric and substance use co-morbidities are at even higher risk of relapse (Leeman et al., 2008; Le Strat et al., 2011; Anthenelli et al., 2016). Although varenicline shows superiority over the other available treatments, the high relapse rates demonstrate the need for new and improved treatment options.

Growing evidence demonstrates the insular cortex (insula) role in nicotine dependence and addiction in general. It was first Naqvi and colleagues who brought the attention to this brain region when they found that individuals who suffered damage to the insula were able to quit smoking immediately, easily and without relapse (Naqvi et al., 2007). Since then, the insula has now become a region of great interest for addiction research. It has been proposed that the insula plays a role in interoception and homeostasis, whereby it integrates internal and external signals to maintain homeostasis (Craig, 2009; Naqvi and Bechara, 2010). In terms of addiction, the insula is crucial in drug seeking behavior, such that individuals can maintain that homeostatic level. It has been linked to a multitude of other functions such as decision making, conscious urges and more (see review Ibrahim et al., 2019). Furthermore, our laboratory has shown inactivation of the insula in rats leads to a significant reduction in nicotine self-administration and seeking after exposure to nicotine associate cues or a priming dose (Forget et al., 2010; Pushparaj and Le Foll, 2015). In addition, modulating the insula’s function via deep brain stimulation also reduced nicotine taking and seeking (Pushparaj et al., 2013). Human neuroimaging studies have also linked the insula to cigarette craving and as a predictor of relapse in smokers (Brody et al., 2002; Jasinska et al., 2014).

Previously, the insula was not an area that was reachable via brain stimulation non-invasively due to it being deeply embedded in the brain. However, with the advancement of technology it is now feasible to stimulate the insula using deep repetitive transcranial magnetic stimulation (rTMS) (Zangen et al., 2005). This technique is relatively painless, does not require anesthesia and is not associated with significant side effects. Depending on the targeted area and the parameters, one can cause facilitation or suppression of neurons beneath the coil (Barker et al., 1985; Lefaucheur et al., 2014). At the time the protocol was written only one study investigating the effect of deep rTMS with an H-coil targeting the insula and dorsolateral prefrontal cortex (DLPFC) for addiction was completed. In fact, it was tested as a treatment for smoking cessation and was found to be effective (Dinur-Klein et al., 2014). Since then, a multi-center smoking cessation trial was completed, which also was found to be effective (Zangen and George, 2020). However, in both studies the prefrontal cortex was targeted along with the insula. There has been one trial investigating deep rTMS to the insula and overlaying regions for alcohol addiction, but it did not find a significant effect (Perini et al., 2020). Thus, it may be specific to nicotine addiction. Given the promising effects of the insula’s role in nicotine addiction and the need for treatments to work long-term, we have developed a study to test the utility of adding insula deep rTMS to varenicline treatment for smoking cessation.

### 1.1 Aims

The primary aim of this study is to examine the efficacy of bilateral deep repetitive transcranial magnetic stimulation (10 Hz) directed to the insular cortex, relative to sham stimulation, on point prevalence smoking abstinence in smokers who are receiving varenicline treatment. The secondary aim is to examine the effect of deep rTMS to the insular cortex on other smoking outcomes such as self-reported craving, cigarette smoking and dependence severity. Lastly, a secondary aim is to examine the effect of deep rTMS to the insular cortex on smoking abstinence at the end of rTMS treatment (i.e., week 4; short term abstinence) and at 6-month follow up (i.e., week 26; long term abstinence).

## 2 Methods

### 2.1 Study design

This study will be a randomized, double-blind, sham-controlled clinical trial. Fifty nicotine dependent participants who have expressed motivation to quit will be randomized into either active (10 Hz, *n* = 25) or sham (*n* = 25) rTMS stimulation. All participants will receive open label varenicline for 12 weeks. All participants will have a target quit date set for Week 2 (Day 15). Participants will all receive weekly counselling sessions from the “Smoke Free and Living It” manual by the Mayo clinic. The rTMS treatment will begin on Week 1 (Day 1) and will be given daily for 4 weeks (5 days/per week). An H-coil targeting selectively the insula will be used for the deep rTMS. After the rTMS intervention is completed, we will have weekly follow-up visits for the duration of the varenicline treatment. Lastly, there will be one follow up visit at Week 26. This trial will be conducted in compliance with the protocol, Good Clinical Practice, and ISO 14155 (Clinical investigation of medical devices).

#### 2.1.1 Participant recruitment

Participants will be recruited through various sources such as the community, clinics at the Centre for Addiction and Mental Health (CAMH), word of mouth, flyers posted in the downtown Toronto area. Ads will also be placed in newspapers, in subways (Toronto Transit Commission), and online (Facebook and Kijiji). All advertisements will be approved by the Research Ethics Boards (REB) prior to use. Recruitment can also be done through similar protocols approved at CAMH provided that the participants have agreed to being contact about future studies.

CAMH also has recruitment programs in place whereby participants may be recruited through members treating the potential participant should they be interested in the study. Also, delegated research coordinators can identify potential participants who have contacted CAMH and put them in touch with the study team. This process is only done with clients who agree to be approached.

The recruitment strategies mentioned are in line with our aim to enroll a representative sample of smokers. This is different than more traditional smoking cessation clinical trials, whereby most smokers are often excluded due to co-morbidities, thus jeopardizing the generalizability of the data (Le Strat et al., 2011). Our exclusion criteria may be regarded as less restrictive for this purpose.

#### 2.1.2 Inclusion criteria

The inclusion criteria for this study are as followed:

1) Age 18–65;
2) Nicotine dependent as assessed by Diagnostic and Statistical Manual of Mental Disorders (DSM-5);
3) Reported daily cigarette consumption ≥10;
4) Expired carbon monoxide (CO) measurement of ≥10 ppm;
5) Fagerström Test of Nicotine Dependence (FTND) ≥ 4;
6) Reported motivation to quit within 30 days as assessed using the Contemplation Ladder score of ≥7.

#### 2.1.3 Exclusion criteria

The exclusion criteria for this study are as followed:

1) Reported smoking abstinence in the 3 months preceding screening visit;
2) Current use of other smoking cessation aids;
3) Allergy and/or contraindication to varenicline or rTMS;
4) Pregnancy, trying to become pregnant or breastfeeding;
5) Current or recent history of cardiovascular or cerebrovascular disease and/or current hypertension;
6) Current or historical evidence of suicidal behavior;
7) Serious current or personal history of medical condition/disease (neurological disorders, brain lesions, multiple sclerosis, head trauma, loss of consciousness, hearing loss, etc.);
8) Current, personal history or family history of seizures;
9) Cognitive impairment as defined as a Mini Mental State Examination (MMSE) score <24;
10) Concomitant use of medication that lowers seizure threshold.

#### 2.1.4 Interventions

##### 2.1.4.1 Varenicline

All participants will receive open label varenicline for 12 weeks. The dosing schedule that will be used is that standard for smoking cessation. It consists of 0.5 mg tablet once a day for the first 3 days, 0.5 mg tablet twice a day (i.e., BID: AM and PM) for the following 4 days. Beginning Week 2 (i.e., Day 8), the target daily dose of 1 mg BID (AM and PM) will be given for the rest of the treatment phase. If, 1 mg BID is not tolerated, 0.5 mg BID may be used. Participants will be advised to orally consume the tablets with food and water. A target quit date for all participants will be set for Day 15. Varenicline will be dispensed weekly by the CAMH pharmacy. We will assess compliance of any missed doses during our weekly follow up visits.

##### 2.1.4.2 Repetitive transcranial magnetic stimulation

Participants will be randomized in a 1:1 ratio to either receive active (10 Hz, *n* = 25) or sham (*n* = 25) rTMS. Brainsway Ltd. (the deep rTMS manufacturer) will provide randomization cards along with the randomization list and it will be managed by the pharmacy and an additional independent person outside of the study. The rTMS treatment will begin on Day 1 and consists of five sessions per week for four continuous weeks (20 sessions total). As previously mentioned, the target quit date will be set to Day 15, which allows for rTMS to be administered for 2 weeks prior and 2 weeks after the quit date. This will hopefully aid smokers since they are most susceptible to relapse during early in the abstinence phase. Both the active and sham stimulation will be administered using the same coil. We are using an H-coil targeting the insula bilaterally by Brainsway Ltd. (Israel, Model 102B). The active stimulation will comprise of 34 trains of 3 s each at 10 Hz and 30 pulses per train and an inter-train interval of 26 s. This is based on a previous smoking cessation study (Dinur-Klein et al., 2014), as well as the input from the TMS experts at the Temerty Centre for Therapeutic Brain Intervention at CAMH. Resting motor threshold (RMT) will be determined during the first treatment by finding the minimum intensity to cause one of the right abductor pollicis brevis muscle to activate in at least 5 of 10 trials. Treatment will then be administered at 120% of RMT. Titration of stimulus intensity may occur over the first four sessions to enhance tolerability. For participants with poor tolerability (measured by a 10-point Likert pain scale) the stimulation target will be a minimum of 110% of RMT. The insula is targeted based on the coordinates of the motor area found during RMT. From the RMT position, the helmet is re-centered and moved anteriorly 6 cm. These coordinates are provided by Brainsway and allow for the helmet to be oriented to the bilateral insula. With regards to the sham stimulation, the same coil placement will be used, and the number of pulses delivered will match those of the active stimulation. The sham coil (built into the same helmet) mimics the acoustics and scalp sensations delivered by the active stimulation. Since rTMS sessions will occur daily for 4 weeks, medical staff will be onsite during all procedures and standard procedures for treating patients with rTMS in the Temerty Centre for Therapeutic Brain Intervention at CAMH will be followed.

#### 2.1.5 Procedures

##### 2.1.5.1 Screening assessment and consent

Potential participants will undergo a telephone pre-screen to determine initial eligibility. Participants may also complete this screening electronically, through REDCap (see REDCap section below). They will subsequently be invited to an in-person screening assessment to confirm final eligibility to participate.

At the start of the in-person screening assessment, the Informed Consent Form will be reviewed, and a quiz will be administered to confirm consent understanding. Upon completion of the review and quiz, the consent form will be signed by both the participant and the study staff obtaining consent.

The following measures and assessments will be administered at the screening visit:

• Fagerström Test of Nicotine Dependence (FTND). The FTND is a measure of physical dependence severity (Heatherton et al., 1991).
• Diagnostic and Statistical Manual of Mental Disorders (DSM-5/SCID-5). The DSM/SCID-5 will be used to diagnose nicotine dependence/tobacco use disorder in participants and/or any other psychiatric issues (American Psychiatric Association, 2013).
• Timeline Followback (TLFB). The TLFB is a reliable method used to detail nicotine (smoking), alcohol and caffeine consumption behavior over a specified period of time. Participants are asked to recall key anchoring events to prompt retrospective estimates of cigarette, alcohol or caffeine intake (Robinson et al., 2014).
• Expired Carbon Monoxide (CO) Measurements. Breath CO measurements provide a reliable indication of recent smoking status among smokers.
• Smoking Contemplation Ladder. This questionnaire allows for the measure of readiness to quit smoking in individuals. A cutoff score of 7 is commonly used to determine individuals who are motivated to quit (Biener and Abrams, 1991).
• Mini Mental State Examination (MMSE). The MMSE is a gross measure of cognitive status in individuals, with a commonly used cutoff of <24. The test will serve as indication of cognitive competence to provide informed consent (Folstein et al., 1975).
• Medical and/or psychiatric assessment by study physician (or delegate).
• Blood draw for quantification of cotinine, complete blood cell count, routine blood chemistry and/or beta-HCG (serum pregnancy test, for females).
• Systemic Assessment for Treatment Emergent Effects (SAFTEE): A technique for the systematic assessment of side effects in clinical trials (Levine and Schooler, 1986).
• Drug screen (urine or blood).
• Demographic and contact forms.
• TASS form (TMS Adult Safety Screening Questionnaire). This questionnaire serves to ensure there are no safety concerns with regards to TMS.

##### 2.1.5.2 Study visits

Other than the screening assessment, there will be a total of 30 study visits. The first study visit corresponds to the first varenicline dose and the first rTMS session. Participant will come in to receive rTMS daily for four consecutive weeks (5 days/week) for a total of 20 visits. Weekly follow-up visits will occur for the duration of the treatment phase (12 weeks total) and a final visit at Week 26. The TLFB, SAFTEE, concomitant medication log and adverse event log will be administered/updated at every visit. CO measurements will be taken at every visit. Urine drug testing and pregnancy testing (for females) will occur once a week at the weekly follow-up visits. Selective questionnaires (described in the Outcome Measures section) will be administered during study sessions. Medication compliance will be monitored at weekly follow-up visits. Suicidality will be assessed weekly at the follow up visits using the Columbia Suicide Severity Rating Scale (Posner et al., 2011). Counselling will be conducted once a week at the weekly follow-up visits. Counselling is clinically recommended to be administered alongside smoking cessation medication for increased efficacy (Anthenelli et al., 2016), a trained study member will deliver brief counselling interventions. The counselling sessions will be adapted from the Mayo Clinic “Smoke Free and Living It,” a commonly used manual in pharmacotherapy trials for nicotine dependence and will cover various topics aimed at building problem-solving skills and providing support through quitting and withdrawal (Croghan et al., 2012). There will be a total of four blood draws: the screening session, end of Week 4, end of Week 12 and at Week 26. Cotinine blood samples will not be analyzed upon collection. The clinical lab will process the samples and the study stuff will pick up the samples and store them in a −80°C freezer. Analysis will be done in batches of 30 or more samples. See Table 1 for summary of study visits.

**TABLE 1:** Summary of events outlining the assessments, measures, and treatments done at each study visit.

	Week and visit number														
			W1		W2		W3		W4		W5	W6–8	W9	W10–12	W13	W26
		1^a^	2	3–6	7	8–11	12	13–16	17	18–21	22	23–25	26	27–29	30	31
Assessments and measures	ICF and ICF quiz	X														
	Contact and Demographic Form	X														
	Medical assessment, SCID, and ECG	X														
	TASS	X														
	MMSE	X														
	Smoking Contemplation Ladder	X														
	Blood draw	X									X				X	X
	CO measurement	X	X	X	X	X	X	X	X	X	X	X	X	X	X	X
	TLFB (Cigarettes, alcohol, caffeine)	X	X	X	X	X	X	X	X	X	X	X	X	X	X	X
	Concomitant medication log	X	X	X	X	X	X	X	X	X	X	X	X	X	X	X
	AE Log	X	X	X	X	X	X	X	X	X	X	X	X	X	X	X
	SAFTEE	X	X	X	X	X	X	X	X	X	X	X	X	X	X	X
	Urine drug test	X	X		X		X		X		X	X	X	X	X	
	Pregnancy test (if required)	X	X		X		X		X		X	X	X	X	X	
	FTND	X	X		X		X		X		X		X		X	X
	Counselling		X		X		X		X		X	X	X	X	X	
	C-SSRS		X		X		X		X		X	X	X	X	X	X
	Medication compliance		X		X		X		X		X	X	X	X	X	
	T-QSU		X		X		X		X		X		X		X	X
	MNWS		X		X		X		X		X		X		X	X
	Point prevalence abstinence										X				X	
	Prolonged abstinence															X
	Continuous abstinence															X
	Quit day						X									
	RMT for rTMS		X													
Treatment	rTMS		X	X	X	X	X	X	X	X						
	Varenicline		X	X	X	X	X	X	X	X	X	X	X	X		

ICF, Informed Consent Form; SCID, Structural Clinical Interview for DSM-5 Disorders; ECG, Electrocardiogram; TASS, TMS Adult Safety Screening Questionnaire; MMSE, Mini Mental State Exam; CO, Carbon monoxide; TLFB, Timeline Followback; AE, Adverse event; SAFTEE, Systemic Assessment for Treatment Emergent Effects; FTND, Fagerström Test of Nicotine Dependence; C-SSRS, Columbia Suicide Severity Rating Scale; T-QSU, Tiffany Questionnaire of Smoking Urges; MNWS, Minnesota Nicotine Withdrawal Scale; RMT, Resting Motor Threshold; rTMS, repetitive Transcranial Magnetic Stimulation.

a Visit 1 corresponds to the eligibility assessment.

During the rTMS treatment phase, urine drug testing, pregnancy testing, counselling, FTND, C-SSRS, T-QSU, MNWS and medication compliance will be done once a week. In the event that a participant misses a session during this time where these assessments were intended to occur, they will be conducted at the following visit. Participants are intended to receive 20 rTMS sessions, however, any missed rTMS sessions (whether due to the participant or to holiday closure) will not be rescheduled.

During the follow up visits (i.e., sessions 22–31), in the event a subject misses a session; they will be able to reschedule the session within the same week. If they are not able to attend within the same scheduled week, the session will be considered as a missed session. The following visits will proceed as scheduled.

#### 2.1.6 Outcome measures

The main outcome measure is a 7-day point prevalence abstinence at the end of 12 weeks (i.e., end of varenicline treatment). This will be measured by using self-report of abstinence in the past 7 days, and confirmed using a plasma cotinine measurement of <15 ng/ml. We have several secondary outcome measures: 1) Fagerström Test of Nicotine Dependence (FTND) (Heatherton et al., 1991). This will be used to assess physical dependence severity; 2) Expired carbon monoxide (CO) measurements. This will be measured regularly as a biological confirmation of recent smoking; 3) Cigarettes per day. The Timeline Followback (TLFB) will be used to determine daily self-reported cigarette consumption (Robinson et al., 2014); 4) Minnesota Nicotine Withdrawal Scale (MNWS). This will be used to assess nicotine withdrawal symptoms (Hughes and Hatsukami, 1986); 5) Tiffany Questionnaire of Smoking Urges (T-QSU). This will be used to assess nicotine craving symptoms (Tiffany and Drobes, 1991); 6) Point prevalence abstinence at end of 4 weeks. This will be measured by self-report of abstinence for the past 7 days and confirmed using plasma cotinine; 7) Prolonged abstinence from end of treatment (Week 12) to end of follow up (Week 26). This outcome will be measured by self-report of continuous abstinence since the last visit (at Week 12) and confirmed using a plasma cotinine; 8) Prolonged abstinence with 2-week grace period at end of follow up (Week 26). This outcome will be measured by self-report of continuous abstinence since Week 4 and confirmed using a plasma cotinine; 9) Continuous abstinence at 6 months. This will be measured by self-report of abstinence since the target quit day (Week 2) and confirmed using plasma cotinine.

**FIGURE 1:**
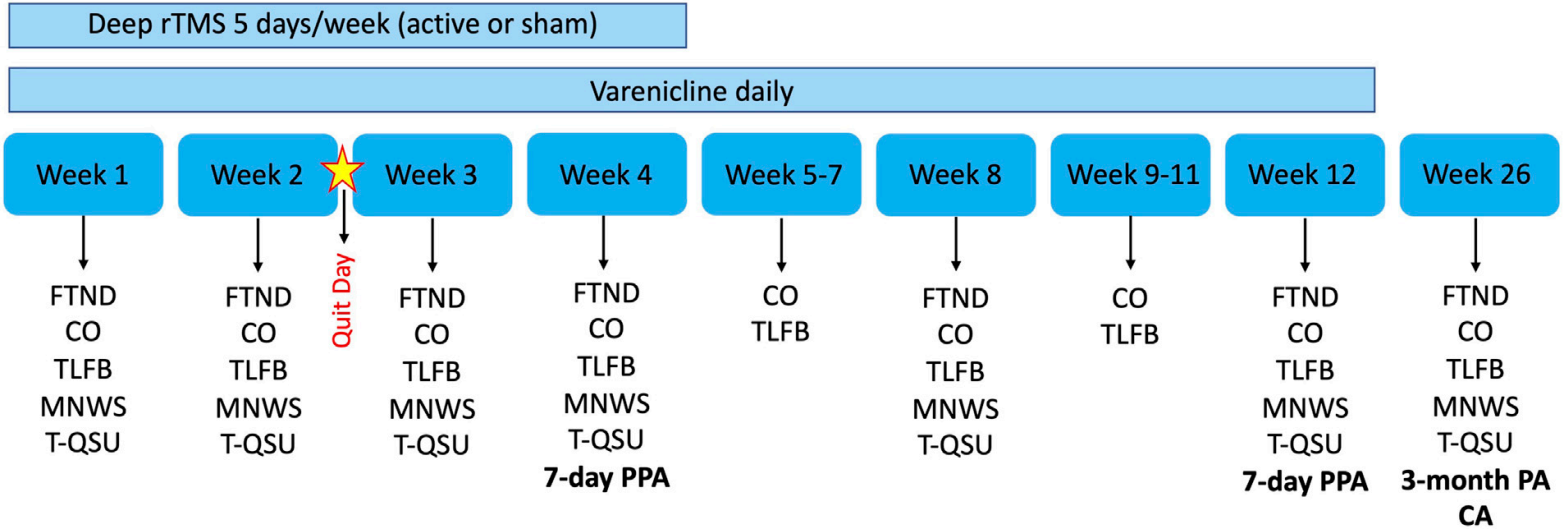
Overview of trial design and outcome measures. Bold indicates our abstinence measures which also includes plasma cotinine measurements. rTMS, repetitive transcranial magnetic stimulation; FTND, Fagerström Test of Nicotine Dependence; CO, Carbon monoxide; TLFB, Timeline Followback; MNWS, Minnesota Nicotine Withdrawal Scale; T-QSU, Tiffany Questionnaire of Smoking Urges; PPA, Point prevalence abstinence; PA, Prolonged abstinence; CA, Continuous abstinence.

**FIGURE 2:**
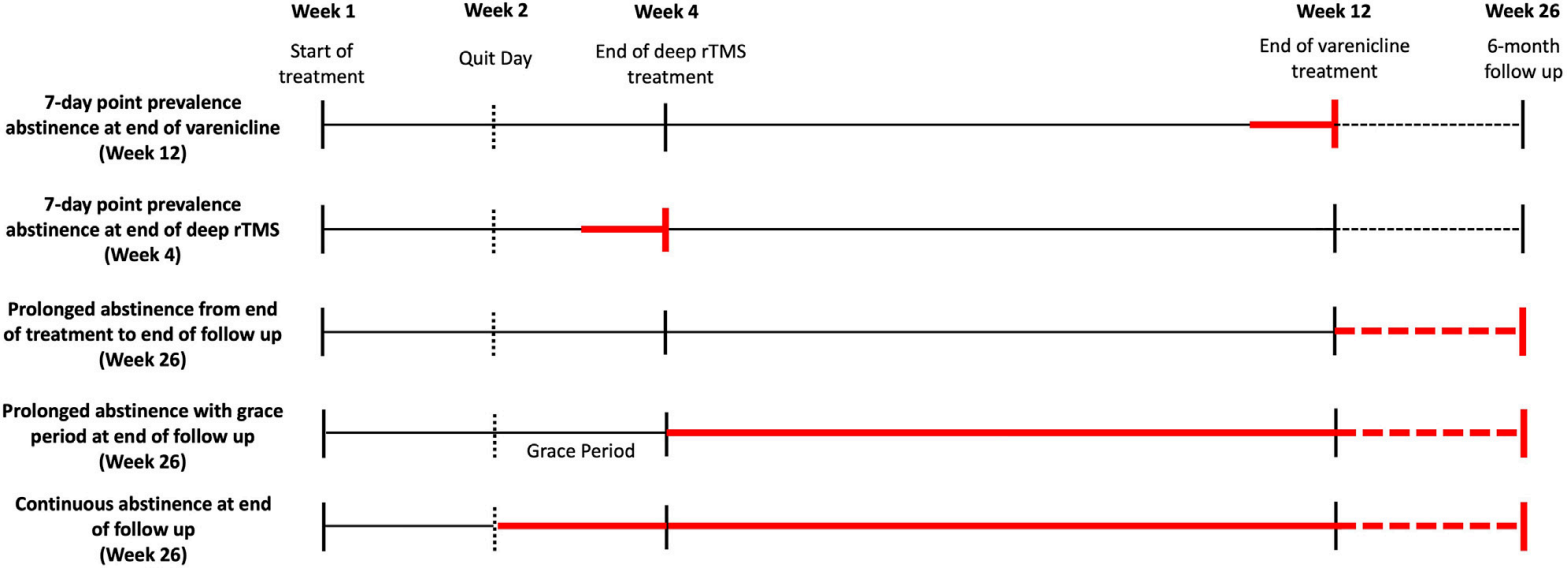
Timeline of Abstinence measures. Abstinence measures are collected at various points during the trial (Week 4, 12, and 26). Measures of various abstinence durations are collected (represented by the red bolded lines). All abstinence measures are collected with self-report and verified with plasma cotinine.

The FTND, T-QSU and MNWS will be administered weekly for the first 4 weeks and subsequently at Week 8, 12, and 26. See Figure 1 for timeline of all outcome measures and Figure 2 for timeline of abstinence measures.

#### 2.1.7 Sample size and power calculations

Based on results from a multi-center trial (recently completed at the time the protocol was written) (Anthenelli et al., 2016) and results from our previous trial investigating varenicline’s efficacy in patients in a residual drug program (Zawertailo et al., 2020), we anticipate that treatment with varenicline will result in a 12-week abstinence point prevalence rate of 40%. In order to detect a clinically relevant 30% difference in abstinence rates between the active deep rTMS and sham treatments with a power of 0.80 (*α* = 0.05), 42 participants per study arm would be needed. However, since this is a pilot study, we will recruit *n* = 25 participants per study arm (power = 0.66). We do anticipate attrition with this kind of study, therefore we will recruit *n* = 30 per arm to reach 50 completers in total.

#### 2.1.8 Data analysis

Descriptive statistics will be used to compare groups (Sham, rTMS) at baseline on main clinical and demographic variables. Fisher’s Exact and Mann-Whitney *U* test will be used for categorical and continuous variables, respectively. Subjects with missing values in the primary outcome (abstinence at 4, 12 or 26 weeks) will be compared with completers on baseline characteristics, to help in the understanding of the reasons for missing values, if at least 10 subjects (20%) are found to be missing. Mixed effect logistic regression using abstinence at 4, 12, and 26 weeks as dependent variables will be adjusted to the data, with groups and categorical time (4, 12, and 26 weeks) specified as fixed effect, and subject intercepts as random effects. Baseline variables correlated with missingness and/or known to be associated with the outcome will be added as covariates with focus on model parsimony (not to include many covariates) due to small sample size. The primary hypothesis will be tested using a contrast that compares abstinence at week 12 in the logit scale. Similarly, abstinence will also be compared at weeks 4 and 26 as part of our secondary objectives. Mixed effect models that uses maximum likelihood estimation accounts for missingness in the dependent variable by using all available information in the estimation, under the MAR assumption (Missing At Random–the values of a data points are not associated with the missing status of the data point after accounting for relevant covariates) (Molenberghs and Verbeke, 2005). Other measures related to our secondary objectives (e.g., FTND, CO, TLFB, etc.) will also be analyzed using linear mixed or generalized mixed models, depending on the nature of the outcome of interest. Graphs will be used to explore the data, the models assumptions (for example, residual plots), as well as model results (for example, estimated means plot). Due to small sample, effect sizes with 95% confidence intervals as well as standardized effect sizes will be reported.

#### 2.1.9 REDCap

REDCap is a research electronic data capture platform tool. This will be used for recruitment purposes should the participant decide to complete the preliminary screening questionnaires electronically. To gain access to the survey, participants will respond to our advertisement by email or telephone. Staff will reply to them with a unique Study ID that the participant will use to gain access to the survey. No personal health information will be stored in the same project as the survey. At the end of the survey participants will give consent to be contacted and they will be directed to a stand-alone project with their telephone number and name. The only link between the survey and telephone number project will be a unique Screening ID.

REDCap will also be used to store data collected during the study visits. Study staff will enter the data collected on paper forms during the visits into the REDCap project, which will then be used to extract the data for analysis.

#### 2.1.10 Participant safety and adverse events

The occurrence of adverse events (AEs) resulting from varenicline treatment and/or rTMS intervention is a possibility. However, in a previous trial completed by our team, we demonstrated the safe use of varenicline in smokers presenting with co-morbid alcohol dependence (Anthenelli et al., 2016; Zawertailo et al., 2020), as well as the safe use of insula deep rTMS in healthy controls (Malik et al., 2018). Regardless, possible side effects will be monitored on a regular basis during the medication phase. Moreover, we will follow current guidelines for the safe use of both varenicline and deep rTMS, including the exclusion of individuals with any known absolute contraindications to either of these treatments.

Every adverse event and observed device deficiency will be recorded. AEs will be assessed at each study visit. All AEs, whether reported by the subject or observed by study staff/investigators, will be recorded in the AE log along with a brief description, start date/resolution date and any action taken. Symptoms related to smoking cessation will not be recorded as AEs. The AE log will be initialed by the qualified investigator, who will make the determination on relationship of the AE to the investigational device/study procedures.

Serious adverse events and device deficiencies meeting Health Canada’s mandatory problem reporting requirements (Part 3, Medical Devices for Investigational Testing Involving Human Subjects) will be reported to Health Canada within the reporting time periods required by Health Canada and/or to REB in accordance with REB’s local reporting requirements and timelines.

#### 2.1.11 Termination of the study

Reasons for withdrawing individual subjects from the study may include one or more of the following:

• Failure to continue to meet inclusion criteria;
• Severe rTMS side effects;
• Major protocol violation;
• Subject lost to follow-up;
• Withdrawal of consent;
• Pregnancy;
• The subject missed more than 3 days of TMS treatment during the treatment trial period (not including holiday closures or COVID related absences)

Notably, any subject may be discontinued from the study at the discretion of the Qualified Investigator if it is deemed to be in the best interest of the subject.

When participants are prematurely withdrawn, they will be replaced by a new participant until the total recruitment aims are met or until the trial is stopped. Data collected until the time of withdrawal may be used in analyses.

## 3 Discussion

Although the current approved smoking cessation therapies do show efficacy. Relapse remains a large problem even after receiving pharmacotherapy intervention (Hajek et al., 2009; Hajek et al., 2013). Thus, there is a dire need to find interventions that allow for long-term smoking abstinence. We are aiming to test the efficacy of a combined treatment using varenicline and deep rTMS to the insula by conducting a randomized, double-blind, sham-controlled clinical trial.

At the time the protocol was written, only one other smoking cessation deep rTMS trial was conducted. Participants were randomized to receive 13 sessions of deep rTMS to the insula and prefrontal cortex (1 Hz, 10 Hz or sham) while presented with smoking cues. The high frequency deep rTMS (10 Hz; 33 trains of 30 pulses, 3 s each, intertrain interval of 20 s) showed increased smoking abstinence at end of treatment (44%) compared to the other groups and at the 6-month follow up (33%) (Dinur-Klein et al., 2014). The trial led to a multi-center trial using high frequency deep rTMS for smoking cessation. Participants received either active (10 Hz; 60 trains of 30 pulses, 3 s each, intertrain interval of 15 s) or sham stimulation daily for 3 weeks followed by weekly for the next 3 weeks. Again, this trial showed positive results whereby active stimulation led to a 28% quit rate compared to 12% for sham stimulation (Zangen et al., 2021). Brainsway Ltd. received FDA approval in August 2020 for the use of the insula and prefrontal cortex H coil for short-term smoking cessation. Even though this is a step forward in finding novel treatments for smoking cessation, it is still limited to short-term efficacy. It also does not demonstrate if it is the insula, the prefrontal cortex or both that are causing this effect, thus, the need for further trials.

Furthermore, we have previously conducted a deep rTMS to the bilateral insula and imaging study on healthy individuals and found that rTMS was well tolerated and did not cause any serious side effects (Malik et al., 2018). Our goal now with this trial is to provide insight as to whether or not it can be useful in a more generalizable group of smokers. Our protocol differs from the previous trials in that we are targeting the bilaterally insula selectively, our treatment duration is longer, and we are using a combined treatment approach. This trial will therefore demonstrate whether a combined treatment presents any advantages. Varenicline is the superior pharmacotherapy currently available in terms of efficacy (Cahill et al., 2016), thus why it was chosen as the adjunct treatment to deep rTMS. However, this might also lead us to have difficulty in finding a clinically significant effect between our groups since the threshold for improvement is lower given that our control group is also receiving treatment (i.e., varenicline). Our sample size also limits us but given that this is a pilot study, it will hopefully shed some insight and possibly lead to a larger trial if successful. In addition, it should be noted that although the coil is designed to target the insula, the H coils are not focal, thus we cannot eliminate the fact that other areas may also be stimulated. Future studies should look to add an imaging component to best understand which brain regions are being affected with stimulation.

## 4 Trial status

The protocol version currently is 7.0 (May 2022). The trial is currently in the data collection phase and anticipates study completion on 31 December 2022.

## References

[B1] American Psychiatric Association (2013). Diagnostic and statistical manual of mental disorders. Virginia, United States: American Psychiatric Association.

[B2] Anthenelli R. M. Benowitz N. L. West R. St Aubin L. McRae T. Lawrence D. (2016). Neuropsychiatric safety and efficacy of varenicline, bupropion, and nicotine patch in smokers with and without psychiatric disorders (EAGLES): A double-blind, randomised, placebo-controlled clinical trial. Lancet 387 (10037), 2507–2520. 10.1016/s0140-6736(16)30272-0 27116918

[B3] Barker A. T. Jalinous R. Freeston I. L. (1985). Non-invasive magnetic stimulation of human motor cortex. Lancet 325 (8437), 1106–1107. 10.1016/S0140-6736(85)92413-4 2860322

[B4] Biener L. Abrams D. B. (1991). The contemplation ladder: Validation of a measure of readiness to consider smoking cessation. Health Psychol. 10 (5), 360–365. 10.1037//0278-6133.10.5.360 1935872

[B5] Brody A. L. Mandelkern M. A. London E. D. Childress A. R. Lee G. S. Bota R. G. (2002). Brain metabolic changes during cigarette craving. Arch. Gen. Psychiatry 59 (12), 1162–1172. 10.1001/archpsyc.59.12.1162 12470133

[B6] Cahill K. Lindson-Hawley N. Thomas K. H. Fanshawe T. R. Lancaster T. (2016). Nicotine receptor partial agonists for smoking cessation. Cochrane Database Syst. Rev. 2016 (5), Cd006103. 10.1002/14651858.CD006103.pub7 PMC6464943 27158893

[B7] Craig A. D. (2009). How do you feel — Now? The anterior insula and human awareness. Nat. Rev. Neurosci. 10 (1), 59–70. 10.1038/nrn2555 19096369

[B8] Croghan I. T. Trautman J. A. Winhusen T. Ebbert J. O. Kropp F. B. Schroeder D. R. (2012). Tobacco dependence counseling in a randomized multisite clinical trial. Contemp. Clin. Trials 33 (4), 576–582. 10.1016/j.cct.2012.02.014 22406192 PMC3361527

[B9] Dinur-Klein L. Dannon P. Hadar A. Rosenberg O. Roth Y. Kotler M. (2014). Smoking cessation induced by deep repetitive transcranial magnetic stimulation of the prefrontal and insular cortices: A prospective, randomized controlled trial. Biol. Psychiatry 76 (9), 742–749. 10.1016/j.biopsych.2014.05.020 25038985

[B10] Folstein M. F. Folstein S. E. McHugh P. R. (1975). Mini-mental state". A practical method for grading the cognitive state of patients for the clinician. J. Psychiatr. Res. 12 (3), 189–198. 10.1016/0022-3956(75)90026-6 1202204

[B11] Forget B. Pushparaj A. Le Foll B. (2010). Granular insular cortex inactivation as a novel therapeutic strategy for nicotine addiction. Biol. Psychiatry 68 (3), 265–271. 10.1016/j.biopsych.2010.01.029 20299008

[B12] Hajek P. Stead L. F. West R. Jarvis M. Hartmann-Boyce J. Lancaster T. (2013). Relapse prevention interventions for smoking cessation. Cochrane Database Syst. Rev. 8, Cd003999. 10.1002/14651858.CD003999.pub4 23963584

[B13] Hajek P. Tønnesen P. Arteaga C. Russ C. Tonstad S. (2009). Varenicline in prevention of relapse to smoking: Effect of quit pattern on response to extended treatment. Addiction 104 (9), 1597–1602. 10.1111/j.1360-0443.2009.02646.x 19686530

[B14] Heatherton T. F. Kozlowski L. T. Frecker R. C. Fagerström K. O. (1991). The Fagerström test for nicotine dependence: A revision of the Fagerström tolerance questionnaire. Br. J. Addict. 86 (9), 1119–1127. 10.1111/j.1360-0443.1991.tb01879.x 1932883

[B15] Hughes J. R. Hatsukami D. (1986). Signs and symptoms of tobacco withdrawal. Arch. Gen. Psychiatry 43 (3), 289–294. 10.1001/archpsyc.1986.01800030107013 3954551

[B16] Ibrahim C. Rubin-Kahana D. S. Pushparaj A. Musiol M. Blumberger D. M. Daskalakis Z. J. (2019). The insula: A brain stimulation target for the treatment of addiction. Front. Pharmacol. 10 (720). 10.3389/fphar.2019.00720 PMC6614510 31312138

[B17] Jasinska A. J. Stein E. A. Kaiser J. Naumer M. J. Yalachkov Y. (2014). Factors modulating neural reactivity to drug cues in addiction: A survey of human neuroimaging studies. Neurosci. Biobehav. Rev. 38, 1–16. 10.1016/j.neubiorev.2013.10.013 24211373 PMC3913480

[B18] Le Foll B. Piper M. E. Fowler C. D. Tonstad S. Bierut L. Lu L. (2022). Tobacco and nicotine use. Nat. Rev. Dis. Prim. 8 (1), 19. 10.1038/s41572-022-00346-w 35332148

[B19] Le Strat Y. Rehm J. Le Foll B. (2011). How generalisable to community samples are clinical trial results for treatment of nicotine dependence: A comparison of common eligibility criteria with respondents of a large representative general population survey. Tob. Control 20 (5), 338–343. 10.1136/tc.2010.038703 21212379

[B20] Leeman R. F. McKee S. A. Toll B. A. Krishnan-Sarin S. Cooney J. L. Makuch R. W. (2008). Risk factors for treatment failure in smokers: Relationship to alcohol use and to lifetime history of an alcohol use disorder. Nicotine Tob. Res. 10 (12), 1793–1809. 10.1080/14622200802443742 19023831 PMC2764010

[B21] Lefaucheur J.-P. André-Obadia N. Antal A. Ayache S. S. Baeken C. Benninger D. H. (2014). Evidence-based guidelines on the therapeutic use of repetitive transcranial magnetic stimulation (rTMS). Clin. Neurophysiol. 125 (11), 2150–2206. 10.1016/j.clinph.2014.05.021 25034472

[B22] Levine J. Schooler N. R. (1986). Saftee: A technique for the systematic assessment of side effects in clinical trials. Psychopharmacol. Bull. 22 (2), 343–381. 3774930

[B23] Malik S. Jacobs M. Cho S. S. Boileau I. Blumberger D. Heilig M. (2018). Deep TMS of the insula using the H-coil modulates dopamine release: A crossover [(11)C] PHNO-pet pilot trial in healthy humans. Brain Imaging Behav. 12 (5), 1306–1317. 10.1007/s11682-017-9800-1 29170944

[B24] Molenberghs G. Verbeke G. (2005). Models for discrete longitudinal data. New York, NY: Spinger.

[B25] Naqvi N. H. Bechara A. (2010). The insula and drug addiction: An interoceptive view of pleasure, urges, and decision-making. New York, NY: Springer-Verlag. 10.1007/s00429-010-0268-7 PMC3698865 20512364

[B26] Naqvi N. H. Rudrauf D. Damasio H. Bechara A. (2007). Damage to the insula disrupts addiction to cigarette smoking. Science 315, 531–534. 10.1126/science.1135926 17255515 PMC3698854

[B27] Perini I. Kämpe R. Arlestig T. Karlsson H. Löfberg A. Pietrzak M. (2020). Repetitive transcranial magnetic stimulation targeting the insular cortex for reduction of heavy drinking in treatment-seeking alcohol-dependent subjects: A randomized controlled trial. Neuropsychopharmacology 45 (5), 842–850. 10.1038/s41386-019-0565-7 31711065 PMC7075882

[B28] Posner K. Brown G. K. Stanley B. Brent D. A. Yershova K. V. Oquendo M. A. (2011). The columbia-suicide severity rating scale: Initial validity and internal consistency findings from three multisite studies with adolescents and adults. Am. J. Psychiatry 168 (12), 1266–1277. 10.1176/appi.ajp.2011.10111704 22193671 PMC3893686

[B29] Pushparaj A. Hamani C. Yu W. Shin D. S. Kang B. Nobrega J. N. (2013). Electrical stimulation of the insular region attenuates nicotine-taking and nicotine-seeking behaviors. neuropsychopharmacology 38, 690–698. 10.1038/npp.2012.235 23249816 PMC3572467

[B30] Pushparaj A. Le Foll B. (2015). Involvement of the caudal granular insular cortex in alcohol self-administration in rats. Behav. Brain Res. 293, 203–207. 10.1016/j.bbr.2015.07.044 26210935

[B31] Robinson S. M. Sobell L. C. Sobell M. B. Leo G. I. (2014). Reliability of the Timeline Followback for cocaine, cannabis, and cigarette use. Psychol. Addict. Behav. 28 (1), 154–162. 10.1037/a0030992 23276315

[B32] Tiffany S. T. Drobes D. J. (1991). The development and initial validation of a questionnaire on smoking urges. Br. J. Addict. 86 (11), 1467–1476. 10.1111/j.1360-0443.1991.tb01732.x 1777741

[B33] World Health Organization (2021). Tobacco. Available: https://www.who.int/news-room/fact-sheets/detail/tobacco.

[B34] Zangen A. George M. (2020). 3-Weeks of prefrontal and insular deep TMS combined with cue-induced craving significantly increases smoking abstinence: Results from an international, multi-site, randomized, sham controlled trial. Biol. Psychiatry 87 (9), S129. 10.1016/j.biopsych.2020.02.348

[B35] Zangen A. Moshe H. Martinez D. Barnea-Ygael N. Vapnik T. Bystritsky A. (2021). Repetitive transcranial magnetic stimulation for smoking cessation: A pivotal multicenter double-blind randomized controlled trial. World Psychiatry. 20 (3), 397–404. 10.1002/wps.20905 34505368 PMC8429333

[B36] Zangen A. Roth Y. Voller B. Hallett M. (2005). Transcranial magnetic stimulation of deep brain regions: Evidence for efficacy of the H-coil. Clin. Neurophysiol. 116 (4), 775–779. 10.1016/j.clinph.2004.11.008 15792886

[B37] Zawertailo L. Ivanova A. Ng G. Le Foll B. Selby P. (2020). Safety and efficacy of varenicline for smoking cessation in alcohol-dependent smokers in concurrent treatment for alcohol use disorder: A pilot, randomized placebo-controlled trial. J. Clin. Psychopharmacol. 40 (2), 130–136. 10.1097/JCP.0000000000001172 32068562

